# Cytokine Gene Expression and Treatment Impact on MRI Outcomes in Jordanian Patients with Multiple Sclerosis

**DOI:** 10.3390/life15060859

**Published:** 2025-05-26

**Authors:** Sawsan I. Khdair, Mohammed Waleed, Alaa M. Hammad, Lubna Al-Khareisha, Tariq Jaber, Majd Ayash, Frank Scott Hall

**Affiliations:** 1 Faculty of Pharmacy, Al-Zaytoonah University of Jordan, Amman 11733, Jordan; mohammadmos93.mw@gmail.com; 2 Department of Pharmacy, Al-Bashir Hospital, Ashrafeya, P.O. Box 10005, Amman 11151, Jordan; alkhreishahlubna@gmail.com; 3 Department of Neurosurgery, Al-Bashir Hospital, Ashrafeya, P.O. Box 10005, Amman 11151, Jordan; dr.tariq-jaber@hotmail.com (T.J.); magdayash@ymail.com (M.A.); 4 Department of Pharmacology and Experimental Therapeutics, University of Toledo, Toledo, OH 43606, USA; frank.hall@utoledo.edu

**Keywords:** cytokines, fingolimod, multiple sclerosis, Jordanians, MRI, relative gene expression

## Abstract

Background: Central nervous system autoimmune disorders, like multiple sclerosis (MS), are chronic conditions where cytokines contribute significantly to the regulation of inflammation. The diagnosis, progression, and treatment effectiveness of MS are assessed through laboratory tests and clinical evaluation, as well as imaging. Method: This study included 40 healthy individuals as a control group and 75 MS patients, divided into two groups: 45 MS patients receiving fingolimod treatment (MSW) and 30 patients taking other medications (MSOs). Blood samples (3 mL) were collected from all participants, and the mRNA relative expression of cytokine genes (*IL-1β*, *TNF-α*, *IL-6*, and *INF-γ*) was measured. Additionally, MRI images of MS patients undergoing fingolimod or other treatments were analyzed. Results: The MSO patient group displayed higher mRNA expressions of *IL-1β*, *TNF-α*, *IL-6*, and *INF-γ* compared to the control group. Furthermore, *TNF-α*, *IL-6*, and *INF-γ* expressions were elevated in the MSO group compared to the MSW group. MRI scans showed significant improvement in MS patients taking fingolimod compared to those receiving other medications. Conclusions: Fingolimod demonstrated greater effectiveness in improving MS patients’ conditions, possibly due to its impact on cytokine expression.

## 1. Introduction

Individuals affected by multiple sclerosis (MS), an inflammatory autoimmune disorder, are usually diagnosed between the ages of 20 and 45 [1]. According to the third edition of the MS ATLAS, the number of MS patients has increased to 2.9 million, with a prevalence of 35.9 per 100,000 people in 2023, which is 26 percent greater than in 2013 [2,3]. Females are more susceptible than males to developing MS worldwide, with an occurrence ratio of 3:1 compared to males [1,3]. The areas of the central nervous system (CNS} affected determined the clinical picture of the disease. The widespread manifestations of MS include muscular weakness and lethargy, optic neuritis, the loss of muscle coordination, and brainstem and spinal cord disorders [4]. While the etiology of the disease is not fully known, several studies have proposed that different factors play a crucial role in MS development, including environmental and genetic factors. Importantly, genetic factors, particularly variations in human leukocyte antigen (HLA) genes, have been recognized as major factors in the etiology of the disease, with strong evidence confirming their role in increasing susceptibility to MS [5,6,7].

MS pathogenesis involves the infiltration of autoreactive T cells with pro-inflammatory activity into the CNS, resulting in inflammatory lesions, demyelination, and plaque formation. Cytokines are proteins that act as chemical messengers, facilitating communication between immune cells. They are produced from different activated immune cells, such as B cells, T cells, and macrophages, in which they work as a cascade of pro-inflammatory signals [1,8]. However, despite the fact that the exact mechanism of MS pathogenesis remains largely unknown, T cells and antigen-presenting cells (APCs) are the main immune cells that are implicated in the inflammatory aspects of the development of MS. APCs supply T cells with their corresponding antigen and establish a cytokine milieu that determines the inflammatory state. Cytokines are employed by T cells in an autocrine fashion to sustain functioning and polarization and to modulate the immune responses of other cellular processes [1,8,9]. This mechanism eventually results in specific cytokine profiles linked with particular T cell types that can compromise the blood–brain barrier, leading to neuronal injury, inflammation, and demyelination in MS [1]. Diagnostic approaches for MS lack sufficient objective biological markers. A recent study applied nuclear magnetic resonance (NMR)-based metabolomics profiling to pinpoint a new biomarker for MS patients [10]. Magnetic resonance imaging (MRI), along with a physical examination, is currently used to confirm an MS diagnosis. Additionally, MRI is essential for tracking disease progression and assessing treatment effectiveness. It can detect lesions in the brain and spinal cord, which are characteristic of MS. To enhance the visibility of these lesions, an intravenous (IV) contrast dye may be administered, which helps to identify when the disease is in an active phase [11].

Multiple clinical studies alongside preclinical studies of experimental autoimmune encephalomyelitis (EAE) have been conducted to highlight the crucial role of cytokines in MS development. Pro-inflammatory cytokines include interleukin-1β (*IL-1β*), tumor necrosis factor-α (*TNF-α*), and interleukin-6 (*IL-6*), while anti-inflammatory cytokines include interleukin-10 (*IL-10*) and interferon-gamma (*INF-γ*), which play essential roles in modulating both the innate and adaptive immune systems [1,8,9,12,13,14,15]. Interpreting the balance of these complex cytokine networks is a challenge facing the development of effective therapeutic strategies for MS [16]. The history of MS therapy over the last 25 years, such as it is, illustrates how this basic research may be successfully translated into therapeutic strategies and enhancements in clinical outcomes. Different medications have been used over the past 32 years based on this understanding. The first injectable medications to be approved for the treatment of MS were interferons (IFNs); this included mainly interferon beta-1b and glatiramer acetate, and then, later, different types of injectable monoclonal antibodies were used. Another milestone in the history of MS therapy was the orally approved medication fingolimod, followed by teriflunomide [1,17]. Fingolimod became the first oral treatment authorized by the U.S. Food and Drug Administration (FDA) for relapsing MS. Its development was influenced by research on modified fungal metabolites and lysophospholipid (LP) sphingosine 1-phosphate (S1P) receptors, which helped clarify this treatment’s mechanism of action and highlighted LP receptors as potential therapeutic targets. Additionally, fingolimod’s direct impact on the central nervous system (CNS) is believed to underlie its efficacy in MS, chiefly in managing progressive disease stages and neurodegeneration [18]. While previous studies have explored the immunological effects of fingolimod in MS patients, most have focused either on serum cytokine levels or clinical outcomes, with limited integration of molecular data and imaging findings. Moreover, only a few studies have directly compared the cytokine expression profiles of patients receiving fingolimod with those of other disease-modifying therapies, particularly in real-world settings. The present study addresses this gap by combining mRNA-based cytokine profiling with MRI results, offering a more comprehensive view of fingolimod’s therapeutic effects on both immune activity and CNS pathology. Additionally, this is the first study of its kind conducted in a Jordanian MS cohort, contributing regionally relevant data to the global literature. By linking transcriptional immune responses with imaging outcomes, this research provides novel insights into the potential mechanisms underlying fingolimod’s effectiveness in treating MS disease outcomes. In the current study, we examine for the first time the effects of fingolimod treatment on MS gene expression signatures using a large panel of inflammatory markers: interleukin-1β (*IL-1β*), tumor necrosis factor-α (*TNF-α*), interleukin-6 (*IL-6*), and interferon-gamma (*INF-γ*). Moreover, this profile is compared to the profiles of other MS treatments in Jordanian MS patients. We also examine the MRI results available for these patients to compare the fingolimod treatment outcomes.

## 2. Methods

### 2.1. Sample Collection

This is a **cross-sectional observational study** with both retrospective and prospective components. Clinical and demographic data were extracted retrospectively from the medical records of patients diagnosed with multiple sclerosis (MS) at Al-Basheer Hospital in Amman, Jordan, between January 2022 and November 2022. In parallel, blood samples were collected prospectively from the same patients during a single hospital visit without any follow-up or intervention.

This study included 40 healthy individuals as a control group and 75 MS patients divided into two groups: 45 MS patients receiving fingolimod treatment (MSW) and 30 MS patients (MSO) receiving alternative treatments, such as beta-interferons, glatiramer acetate, or intravenous immunoglobulin. All of the participants were Jordanians who were treated at Al-Basheer Hospital in Amman, Jordan. The diagnosis of MS patients was performed according to McDonald’s diagnostic criteria [17,19]. After the volunteers signed a consent form, a questionnaire about their age, gender, and medical history was completed by the investigator and three milliliters of blood were collected from each volunteer in EDTA tubes. Inclusion criteria included a confirmed diagnosis of MS, age ≥ 18 years, Jordanian nationality, and ability to provide informed consent. Exclusion criteria included non-Jordanian nationality and consanguinity among MS patients. The study collected data on various demographics, including age, gender, age at disease onset, smoking status, and medications, as shown in the result section. This study was approved by the ethical commission at the Ministry of Health in Amman, Jordan, The Institutional Review Board (IRB) approval number was 263/2021, and the approval was dated 19 December 2021.

### 2.2. Sample Size Calculation

The sample size was calculated using the Cochran formula for cross-sectional studies to estimate proportions in a population. Based on the reported prevalence of multiple sclerosis (MS) of 39 per 100,000 individuals (i.e., *p* = 0.00039) in Jordan [20], and using a 95% confidence level (*Z* = 1.96) with a desired margin of error of 0.005 (*d* = 0.5%), the required sample size was calculated as follows [21]:

$$n=\frac{Z^{2}\times p\times (1-p)}{d^{2}}=\frac{{\left(1.96\right)}^{2}\times 0.00039\times (1-0.00039)}{{\left(0.005\right)}^{2}}\approx 60$$

Therefore, a minimum of 60 MS patients was determined to be sufficient for the study to estimate population parameters with acceptable precision. To increase the robustness of the analysis and account for potential data loss or exclusions, the final targeted sample size was set at 75 patients.

### 2.3. Extraction of RNA and Synthesis of Complementary DNA

Total RNA was isolated from fresh blood samples utilizing the RNA purification kit (Jena Bioscience, Cat# PP-210S, Jena, Thuringia, Germany) in line with the manufacturer’s protocol. To summarize, 100 μL of anticoagulated blood was transferred into a microcentrifuge tube, mixed with 500 μL of a lysis buffer containing 2-ME, and vortexed for 10 s. The lysed samples were then loaded onto spin columns within a collection tube; afterward, 700 μL of a blood-washing buffer was added. The DNase I and DNA digestion buffer were used to remove genomic DNA contamination from the samples. Sequential washes with RNA prewash and RNA wash buffer were performed in order to obtain purified RNA, followed by extraction with 50 μL of DNase/RNase-free water via centrifugation. Finally, the RNA concentration was measured using a Nanodrop DNA/Protein Analyzer (Quawell, Sunnyvale, CA, USA).

All RNA samples were reverse-transcribed into single-stranded cDNA using the FIREScript RT cDNA Synthesis Kit (Solis BioDyne OÜ, Tartu, Estonia) following the manufacturer’s guidelines. The cDNA synthesis was conducted using a DNA Engine^®^ Peltier thermal cycler (Bio-Rad, Hercules, CA, USA, USA). The total reaction mixture, with a volume of 10 μL, included 2 μL of RNA, 2 μL of reverse transcriptase (RT), and 6 μL of nuclease-free water. The Nanodrop DNA/Protein Analyzer (Quawell) was used to measure the concentration of the synthesized cDNA and adjusted with nuclease-free water to achieve a final concentration of 80–100 ng/μL.

### 2.4. Real-Time, Quantitative Polymerase Chain Reaction Procedure (q-PCR)

The RT-PCR analysis of cytokine genes (*IL-1β*, *TNF-α*, *IL-6*, and IFN-γ) was conducted using SYBR Green detection (TB Green Premix Ex Taq II, Takara Bio, Kusatsu, Japan) on a Prime Pro 48 Real-Time PCR system (Cole-Parmer, St. Neots, UK). The total reaction volume for the quantitative RT-PCR was 20 μL, including 1 μL of diluted cDNA, 1 μL each of forward and reverse primers, 10 μL of SYBR Green, and 7 μL of nuclease-free water. Table 1 lists the primer sequences for the target pro-inflammatory genes, as previously reported. β-actin served as the housekeeping gene for normalization. The 2^−ΔΔCT^ method was used to calculate the relative mRNA expression [22]. Each sample was tested in triplicate.

### 2.5. Magnetic Resonance Imaging

A random subset of patients was selected for magnetic resonance imaging (MRI) from both patients receiving fingolimod as their treatment, and MS patients taking other treatments. The MRI scans were conducted using a Siemens 1.5 Tesla device, and all images were captured with a 3 mm contiguous FLAIR (fluid-attenuated inversion recovery) sequence. A clinician who was blinded to the treatment given to the patients assessed and measured the number of lesions and the size of each lesion in the MRI scan.

### 2.6. Statistical Analysis

All data analysis was conducted using SPSS version 25. Demographic data, including age, gender, and smoking status, were not distributed normally; therefore, non-parametric analysis was performed for the samples, including the independent-samples Mann–Whitney U test as an alternative to the independent samples *t*-test. The mRNA relative expression data for various cytokines were presented as the mean ± SD. Means were compared using one-way analysis of variance (ANOVA), followed by Tukey’s multiple comparisons test to identify significant differences between groups. A *p*-value of less than 0.05 was considered statistically significant.

## 3. Results

There are two main groups in this study: a control group (*n* = 40) and MS patients (*n* = 75). The mean age of the control group was 43.6 ± 13.58 years; the smoking percentage was 70%; and the male-to-female ratio was 1:2. For the MS group, the mean age was 37.77 ± 9.92 years; the smoking percentage was 14.7%; and the male-to-female ratio was (1:2), as shown in Table 2. A Mann–Whitney test revealed that there was no significant difference in the demographic data between the control and MS groups for any variable except age (U = 1170, *p* = 0.03) and smoking status, *p* < 0.0001.

The MS group was subdivided into two drug treatment groups: MS patients treated with fingolimod (MSW) and MS patients treated with other medications, such as beta-interferons, glatiramer acetate, and intravenous immunoglobulins (MSOs). The mean age of the MSW group was 36.2 ± 9.53 years; the mean age at disease onset was 31.16 ± 10.66 years; and the male-to-female ratio was (1:2.33). While the mean age of MSO was 38.82 ± 10.14 years, the mean age of disease onset was 35.53 ± 10.25 years, and the male-to female ratio was (1:1.81), as shown in Table 3. There were no significant differences in these variables.

### 3.1. mRNA Relative Expression of IL-1β, TNF-α, IL-6, and INF-γ in Control, MSW, and MSO Groups

**Figure 1:**
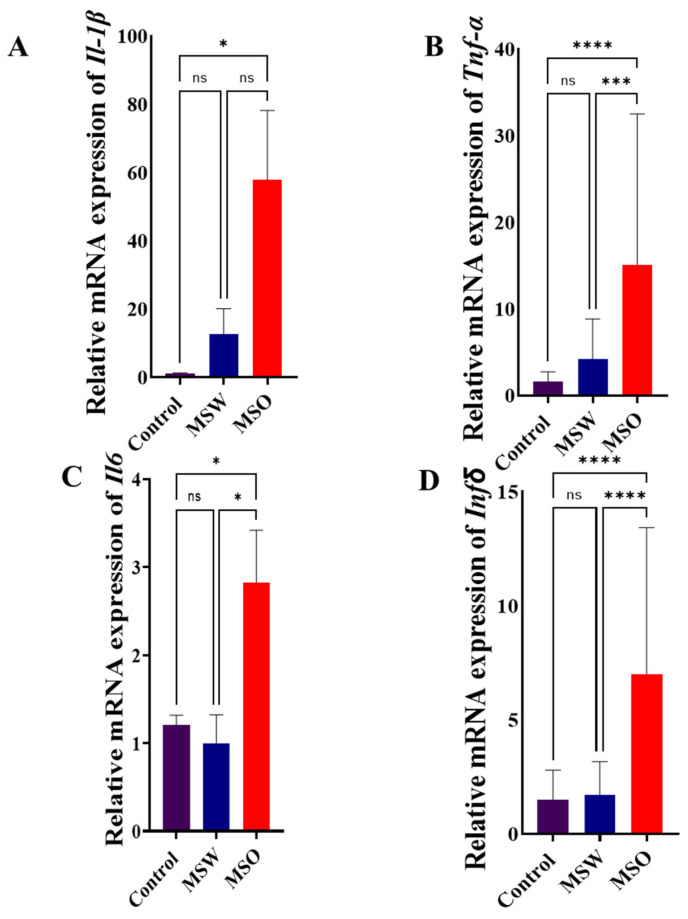
Relative mRNA expression of (**A**) *IL-1β*, (**B**) *TNF-α*, (**C**) *IL-6*, and (**D**) *INF-γ* in control, MSW, and MSO groups. Results are expressed as the mean ± SD (*n* = 40 for control; 30 for MSW and 45 for MSO). (ns: no significance; * *p* < 0.05; *** *p* < 0.001; ****: *p* < 0.0001). Significant differences were based on one-way ANOVA followed by Tukey’s multiple comparisons. The MSO group showed higher mRNA expressions for all measured cytokines compared to the control group, while the MSO group showed higher mRNA expressions for *TNF-α*, *IL-6*, and *INF-γ* compared to the MSW group.

Relative mRNA expressions of *IL-1β*, *TNF-α*, *IL-6*, and *INF-γ* in MS patients treated with fingolimod, MS patients treated with other medications, and control subjects are shown in Figure 1. These results show that the relative expression of *IL-1β* is significantly higher in the MSO group compared to the control group (Figure 1A). Similar results were seen for the mRNA relative expression of *TNF-α*. Here, the MSO group had significantly higher relative mRNA expressions of *TNF-α* compared to both the control and MSW groups (Figure 1B). Similarly, the MSO group had significantly higher relative mRNA expressions of *IL-6* compared to both the control and MSW groups (Figure 1C). Finally, the MSO group had significantly higher relative mRNA expressions of *INF-γ* compared to the control and MSW groups (Figure 1D). These differences were shown using one-way ANOVA for *IL-1β* [F (2, 112) = 4.702, *p* = 0.0113], *TNF-α* [F (2, 112) = 17.24, *p* < 0.0001], *IL-6* [F (2, 112) = 5.664, *p* = 0.0046], and *INF-γ* [F (2, 112) = 22.87, *p* < 0.0001]. The individual mean comparisons noted above were identified by Tukey’s multiple comparisons (*p* < 0.05). No statistical significance in relative mRNA expression between the MSW and control groups was seen for any cytokine measured.

### 3.2. Magnetic Resonance Imaging Results

**Figure 2:**
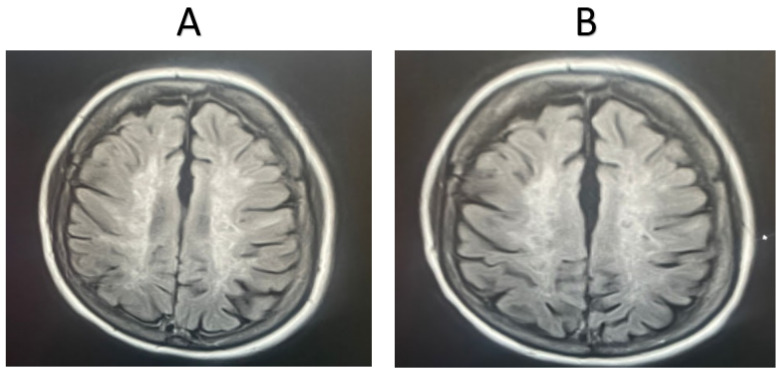
MRI examination of an MSW patient taking fingolimod. (**A**). The number of lesions before treatment = 13, with an average lesion size (cm) of 0.36 ± 0.21, (**B**). The number of lesions after treatment = 6, with an average lesion size (cm) of 0.17 ± 0.24. The number and extent of the lesions were reduced after one year of fingolimod treatment.

**Figure 3:**
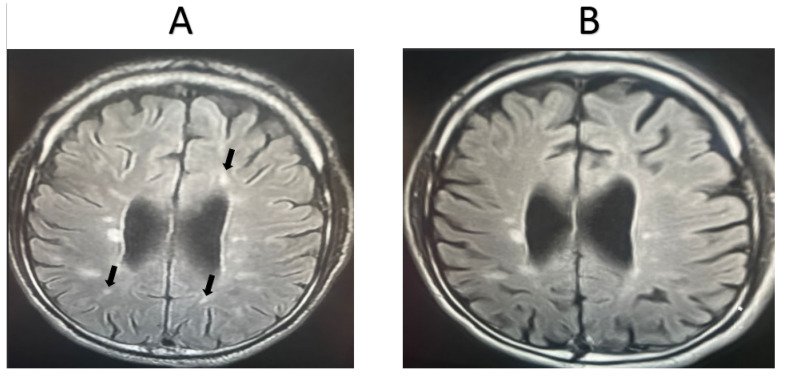
MRI examination of a second MSW patient taking fingolimod. (**A**). The number of lesions before treatment = 13, with an average lesion size (cm) of 0.48 ± 0.24, (**B**). The number of lesions after treatment = 10, with an average lesion size (cm) of 0.37 ± 0.20. The number and extent of lesions were similar after one year of fingolimod treatment (stable). Arrows refer to lesions.

MRI was completed for 11 random patients from the MSW group. In total, 72.72% of the cases showed signs of improvement based on the clinician’s assessment, while 27.28% showed signs of worsening pathology. MRI scans were also taken for 11 random patients from the MSO group. Only 18.18% of these cases showed signs of improvement based on the clinician’s assessment, while 81.82% showed signs of worsening pathology. Representative MRI images from two randomly selected patients receiving fingolimod treatment are shown in Figure 2 and Figure 3. For the first patient, an image taken before fingolimod treatment (Figure 2A) and an image taken after one year of treatment (Figure 2B) are presented. The number of lesions before treatment was 13, with an average size (cm) of 0.36 ± 0.21, while the number of lesions after treatment decreased to 6 with an average size (cm) of 0.17 ± 0.24, indicating an improvement in the patient. In contrast, an image taken before fingolimod treatment (Figure 3A) and an image taken after one year of treatment are presented for another patient (Figure 3B). The number of lesions before treatment was 13, with an average size (cm) of 0.48 ± 0.24, while the number of lesions after treatment decreased to 10 with an average size (cm) of 0.37 ± 0.20, indicating the stable progression of the disease. A complete quantitative description of the number of lesions and their sizes is available in Supplementary Data.

**Figure 4:**
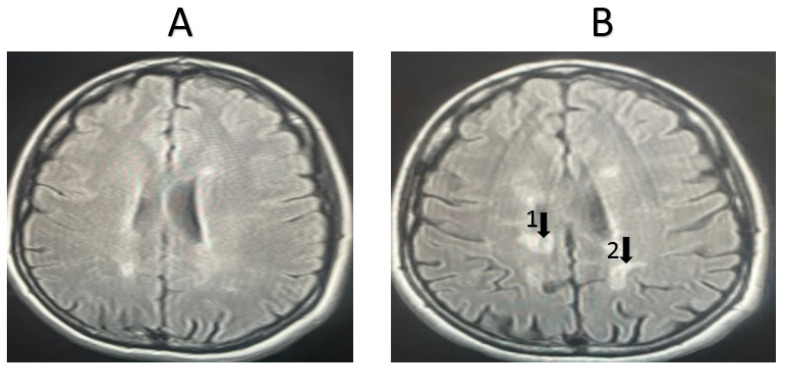
MRI examination of an MSO patient taking another medication (not fingolimod). (**A**). The number of lesions before treatment = 14, with an average lesion size (cm) of 0.35 ± 0.17, (**B**). The number of lesions after treatment = 18, with an average lesion size (cm) of 0.40 ± 0.19. The progression of the disease worsened after one year of treatment. Arrows refer to new lesions formed.

**Figure 5:**
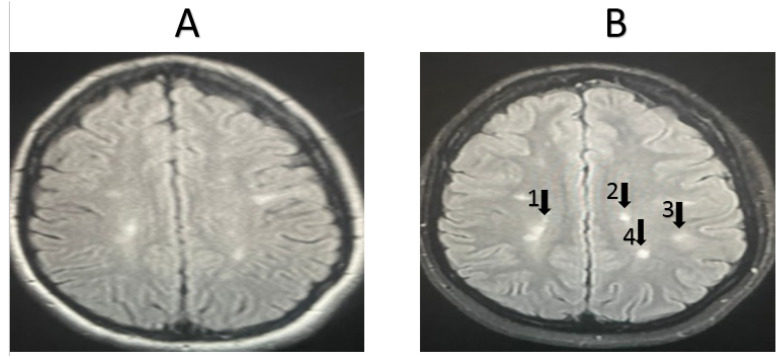
MRI examination of an MSO patient taking another medication (not fingolimod). (**A**). The number of lesions before treatment = 17, with an average lesion size (cm) of 0.45 ± 0.23, (**B**). The number of lesions after treatment = 21, with an average lesion size (cm) of 0.44 ± 0.15. The progression of the disease worsened after one year of treatment. Arrows refer to new lesions formed.

Representative MRI images from randomly selected patients receiving other medications are shown in Figure 4 and Figure 5. For the first patient, the image taken before treatment (Figure 4A) and following one year of treatment (Figure 4B) show an increase in lesion size, indicating the worsening of the condition. Additionally, another set of images for a second patient, taken before treatment (Figure 5A) and after one year of treatment (Figure 5B), also show an increase in lesion size, the appearance of new lesions, and increased lesion intensity, indicating significant disease progression.

## 4. Discussion

This study found that the relative mRNA expressions of *IL-1β*, *TNF-α*, *IL-6*, and *INF-γ* were higher in the MSO patient group compared to the control group. Moreover, the expressions of *TNF-α*, *IL-6*, and *INF-γ* were higher in the MSO patient group than in the MSW group. *IL-1β* is a pleiotropic pro-inflammatory cytokine that plays a role in many inflammatory diseases, including MS [24]. The elevated mRNA expression in the MS patient group compared to the control group in our study aligns with a previous study conducted in Iran, reporting significantly higher *IL-1β* mRNA expression levels in MS patients than in age- and sex-matched controls [24,25]. In addition, our results are compatible with several studies showing increased *IL-6* expression in MS patients in comparison to healthy controls [26,27]. Studies on *TNF-α* expression in MS patients have found that *TNF-α* mRNA expression is significantly higher in individuals with MS compared to healthy subjects [1,28], which is consistent with our findings here. *INF-γ* is a key cytokine known to increase activity in MS and EAE by promoting the production of T helper-1 cells [29]. Moreover, elevated *INF-γ* production induces the release of pro-inflammatory cytokines such as *TNF-α* and *IL-6*, which may explain the increased mRNA expressions of *TNF-α*, *IL-6*, and *INF-γ* observed in the MSO patient group compared to the control group. Additionally, increased *INF-γ* production enhances the expression of *INF-γ* receptor 1 (*INF-γ* R1) on astrocytes in the CNS, leading to astrocyte apoptosis. Overall, suppressing IFN-γ signaling in astrocytes may help mitigate secondary inflammatory infiltration and disease progression [30].

Of critical importance for evaluating the potential clinical value of the treatments assessed here, the relative expressions of *IL-1β*, *TNF-α*, *IL-6*, and *INF-γ* in MS patients taking fingolimod (the MSW group) were substantially lower than those of patients taking other medications (the MSO group). This finding is consistent with a study that found the decreased production of *IL-6* and *TNF-α* as a result of the fingolimod treatment of mature dendritic cells. This effect diminishes the ability of dendritic cells (DCs) to stimulate an inflammatory reaction by T cells in vitro [31]. Our results are also consistent with a previous study that demonstrated that the administration of fingolimod led to a reduction in the secretion of IL-17 with IFN-γ, or IFN-γ on its own, which has critical immunomodulatory effects. Additionally, IFN-γ can restrict the expansion of activated T cells by triggering their apoptosis [30,32,33]. These findings offer a mechanistic explanation for the protective role of IFN-γ in EAE.

The reversal of immune status by fingolimod treatment is consistent with the clinical outcomes in the same patients. Fingolimod was more successful than other treatments in terms of enhancing clinical outcomes as measured by an MRI examination of characteristic brain lesions. This enhancement in the clinical outcome with fingolimod over the other treatments could be related to the decreased mRNA expression for pro-inflammatory cytokines. These findings are similar to another study, which demonstrated that the administration of fingolimod led to significant and long-lasting reductions in brain lesions. Consistent with this outcome, when compared with a placebo, the approved dosage of fingolimod, which is 0.5 milligrams, showed a substantial decrease in brain volume decline [34]. In addition, the results of the current study are consistent with a previous study, which reported better outcomes based on MRI assessments in patients taking fingolimod compared to patients taking other medications [35,36,37]. However, another study demonstrated that there is no difference between fingolimod and natalizumab in terms of MRI outcomes [29]. Other studies also established that natalizumab was more effective than fingolimod when the MRI outcomes were compared [32,33]. Obviously, more studies are needed to determine when and why some treatments produce better outcomes compared to others.

All in all, the better outcomes shown by fingolimod treatment here, through MRI assessments of brain lesions, might be due to fingolimod’s unique mechanisms of action. These include the modulation of S1P receptors, which causes redistribution and decreases in lymphocytes [38], and changes in T and B cell trafficking [39]. Fingolimod modulates S1P1 and S1P3, which are expressed by reactive astrocytes that may cause demyelination, pro-inflammatory cytokine secretion (i.e., *TNF-α*), oligodendrocyte death, and neuronal damage [40]. Fingolimod also modulates S1P1 and S1P5, which are expressed by oligodendrocytes, producing a protective effect and the prevention of oligodendrocyte death [41]. These effects of fingolimod are different from other treatments, like INF beta-1α, which decreases the secretion and migration of T lymphocytes to the CNS and changes metalloproteinase expression [42]. The effect of fingolimod also differs from other immunosuppressive treatments for MS like monoclonal antibodies; this includes the anti-α4β1 monoclonal antibody, which blocks the molecular interaction between α4β1 and vascular cell adhesion molecule-1 (VCAM-1), thus lowering lymphocyte egress to the CNS [43]. Furthermore, monoclonal antibody treatment reduces the release of immunoglobulin types G and M, thereby suppressing inflammation [44,45].

This study provides novel insights into the immunomodulatory effects of fingolimod in MS by examining the mRNA expression levels of cytokines and correlating these molecular markers with imaging outcomes. While fingolimod is a well-established disease-modifying therapy (DMT) known for its sphingosine-1-phosphate receptor modulation, its impact on immune pathways at the molecular level—particularly in relation to MS progression and neuroimaging results—remains incompletely understood. Our findings demonstrate that fingolimod treatment is associated with a significant reduction in the expression of pro-inflammatory cytokines compared to other MS therapies, in addition to showing improved MRI outcomes. This suggests a potential mechanistic link between fingolimod’s immunosuppressive effects and its ability to reduce neuroinflammation and promote neuroprotection, as indicated by MRI findings.

The primary research gap addressed by this study lies in the relatively limited data that directly connect immune modulation, specifically cytokine expression, with MRI-based markers of disease activity in MS patients. Most existing studies focus either on the clinical outcomes of treatments or on isolated immune or imaging parameters. By integrating both molecular and imaging data, our study offers a more comprehensive understanding of fingolimod’s therapeutic effects, bridging a critical gap in the literature. This dual approach to cytokine profiling and MRI analysis provides important insights into how immune responses may influence the progression of MS lesions and other MRI features, such as lesion burden and brain atrophy.

Our study also adds to the body of research by examining a cohort of MS patients in Jordan, contributing to a more diverse understanding of MS therapy outcomes beyond predominantly Western or Eastern cohorts. Given regional differences in genetic, environmental, and healthcare factors, our findings have important implications for treatment strategies in the Middle Eastern context and other similarly affected populations.

Despite the promising findings presented here, several limitations must be acknowledged. First, this study was cross-sectional, meaning that it assessed the relationship between cytokine expression and MRI results at a single time point, limiting the ability to establish causality or observe long-term treatment effects. Longitudinal studies with repeated cytokine measurements and MRI scans over time could provide a more robust understanding of the temporal relationship between fingolimod treatment, immune modulation, and MRI outcomes. Additionally, there are numerous other inflammatory mediators that could have influenced the disease progression of MS, such as IL-17 [46]. Further studies on other biochemical and genetic markers associated with MS are required in the future to clarify the link between MS disease and these markers. This more detailed view might then lead to treatments based on genetic profiles and other markers, eventually leading to individualized treatments and overall improvements in MS treatments. Moreover, expanding the cytokine profile to include additional markers of immune activation and regulation may provide a more comprehensive picture of fingolimod effects. Furthermore, the relatively small sample size, although sufficient for detecting the trends we observed, limits the generalizability of our results and warrants replication in larger, more diverse cohorts. Furthermore, research into the potential use of cytokine expression as a predictive biomarker for long-term clinical outcomes, such as disability progression and relapse rates, is warranted. Larger, multicenter studies involving diverse patient populations could strengthen the generalizability of the findings and help elucidate whether these results are consistent across different MS subtypes and demographics. Moreover, another limitation of this study is the relatively small sample size for MRI analysis, which may limit the statistical power to detect subtle changes in MRI outcomes, such as lesion progression or brain atrophy. A larger sample size would provide greater confidence in the observed MRI improvements and allow for more robust subgroup analyses to assess treatment effects across different MS phenotypes.

Finally, integrating cytokine profiling with other molecular techniques, such as proteomics, may offer even more detailed insights into the cellular and molecular pathways affected by fingolimod and other MS therapies.

## 5. Conclusions

The relative mRNA expressions of *IL-1β*, *TNF-α*, *IL-6*, and *INF-γ* cytokines were elevated in patients with MS. Patients taking fingolimod expressed lower relative mRNA expressions of these cytokines compared to patients taking other treatments, the levels of which were not different from control subjects. Furthermore, better results in MRI-based analyses of brain pathology for patients taking fingolimod were seen compared to other treatments that may be attributed to observed reductions in the cytokine mRNA expressions produced by fingolimod.

## Figures and Tables

**Table 1:** Primer sequences.

Gene	Primer	Sequence
*IL-1β*	F	5′-CCACAGACCTTCCAGGAGAATG-3′
	R	5′-GTGCAGTTCAGTGATCGTACAGG-3′
*TNF-α*	F	5′-CTC TTC TGC CTG CTG CAC TTT G-3′
	R	5′-ATG GGC TAC AGG CTT GTC ACT C-3′
*IL-6*	F	5′-AGA CAG CCA CTC ACC TCT TCA G-3′
	R	5′-TTC TGC CAG TGC CTC TTT GCT G-3′
*INF-γ*	F	5′-TGT AGC GGA TAA TGG AAC TCT TTT-3′
	R	5′-AAT TTG GCT CTG CAT TAT T-3′
*β-actin*	F	5′-TAA TGT CAC GCA CGA TTT CCC-3′
	R	5′-TCA CCG AGC GCG GCT-3′

*IL-6* primer sequence [9]. *INF-γ* primer sequence [17] *TNF-α* and *IL-1β* primer sequence (OriGene Technologies, Inc., MD, USA). β-actin primer sequence [23]. F: forward primer. R: reverse primer.

**Table 2:** Demographic data for control and MS patients.

Parameters		Controls (N = 40) N (%)	MS Patients (N = 75)	Independent Mann–Whitney Test (*p* Value)	Dose	Duration (Years)
Age (years) (mean ± SD)		43.6 ± 13.58	37.77 ± 9.92	*p* = 0.03		
Gender	Male	18 (45%)	25 (35.6%)	ns		
	Female	22 (55%)	50 (64.4%)	ns		
Age at disease onset (years) (mean ± SD)		¯	33.78 ± 10.56	ns		
Smoking Status	Smoker	28 (70%)	11 (14.7%)	*p* < 0.0001		
	Non-smoker	12 (30%)	64 (85.3%)	ns		
Medication	Fingolimod	¯	30 (40%)	ns	0.5 mg daily po	4 ± 1
	Natalizumab	¯	10 (13.3%)	ns	300 mg iv	3 ± 2
	interferon beta-1	¯	19 (25.3%)	ns	144 mcg 3 times a week	4 ± 2
	Others (Ocrelizumab)	¯	16 (21.4%)	ns	600 mg iv	5 ± 2

N = number, % frequency, ns=non-significant.

**Table 3:** Demographic data for the MS patient subgroups: MS patients treated with fingolimod (MSW) and MS patients treated with other medications (MSO). ns denotes a non-significant comparison.

Parameters		MS Patients Taking Fingolimod (MSW) (N = 30)%	MS Patients Not Taking Fingolimod (MSO) (N = 45)%	Independent Mann–Whitney Test (*p* Value)
Age (years) (mean ± SD)		36.2 ± 9.53	38.82 ± 10.14	ns
Gender	Male	9 (30%)	16 (35.6%)	ns
	Female	21 (70%)	29 (64.4%)	ns
Age of disease onset (years) (mean ± SE)		31.16 ± 10.66	35.53 ± 10.25	ns
Smoking Status	Smoker	5 (16.7%)	6 (13.3%)	ns
	Non-smoker	25 (83.3%)	39 (86.7%)	ns

N = number, % frequency, ns = non-significant.

## Data Availability

Data is contained within the article or Supplementary Material.

## Supplementary Materials

The following supporting information can be downloaded at https://www.mdpi.com/article/10.3390/life15060859/s1. Table S1. Quantitative description of number of lesion and size for Figure 2. Table S2. Quantitative description of number of lesion and size for Figure 3. Table S3. Quantitative description of number of lesion and size for Figure 4. Table S4. Quantitative description of number of lesion and size for Figure 5.
