# Supplementary material for: Cytokine Gene Expression and Treatment Impact on MRI Outcomes in Jordanian Patients with Multiple Sclerosis
Source: Life (Basel). 2025 May 26;15(6):859. doi: 10.3390/life15060859 (PMC12193738; doi:10.3390/life15060859)
Supplement: Supplementary file 1 [file life-15-00859-s001.zip › life-3613418-supplementary.pdf]

Supplementary Data

Table S1. Quantitative description of number of lesion and size for Figure 2

**Figure 2**	**Number of lesion**	**Size of lesions (cm)**	**After tx MSW**
**Before tx**	1	0.42	0.3
	2	0.18	0.38
	3	0.25	0.78
	4	0.42	0.21
	5	0.24	0.5
	6	0.18	0.09
	7	0.24	
	8	0.36	
	9	0.5	
	10	1	
	11	0.36	
	12	0.24	
	13	0.25	
	**Mean**	0.356923077	0.173846154
	**SD**	0.209114787	0.240529495

Table S2. Quantitative description of number of lesion and size for Figure 3

**Figure 3**	**Number of lesion**	**Size of lesions (cm)**	**After tx MSW**
**Before tx**	1	0.6	0.66
	2	0.3	0.66
	3	0.44	0.36
	4	0.3	0.66
	5	0.3	0.36
	6	0.3	0.38
	7	0.42	0.85
	8	0.48	0.38
	9	0.72	0.28
	10	0.75	0.2

	11	0.6	0
	12	0.03	0
	13	0.96	0
	**Mean**	0.476923077	0.368461538
	**SD**	0.236460588	0.20032224

Table S3. Quantitative description of number of lesion and size for Figure 4

**Figure 4**	**Number of lesion**	**Size of lesions (cm)**	**After tx MSO**
**Before tx**	1	0.3	0.38
	2	0.3	0.19
	3	0.3	0.3
	4	0.3	0.3
	5	0.4	0.19
	6	0.5	0.2
	7	0.3	0.19
	8	0.5	0.28
	9	0.5	0.28
	10	0.65	0.8
	11	0.74	0.56
	12	0.18	0.48
	13	0.7	0.38
	14	0.7	0.3
	15		0.47
	16		0.77
	17		0.47
	18		0.71
	**Mean**	0.353888889	0.402777778
	**SD**	0.178115131	0.192434853

Table S4. Quantitative description of number of lesion and size for Figure 5

**Figure 5**	**Number of lesion**	**Size of lesions**	**After tx MSO**
**Before tx**	1	0.36	0.47
	2	0.36	0.47
	3	0.54	0.3
	4	0.95	0.39
	5	0.36	0.94

	6	0.72	0.38
	7	0.96	0.48
	8	0.72	0.57
	9	0.36	0.3
	10	0.4	0.59
	11	0.54	0.48
	12	0.65	0.48
	13	0.92	0.48
	14	0.74	0.42
	15	0.36	0.3
	16	0.18	0.28
	17	0.4	0.47
	18	0	0.38
	19	0	0.3
	20	0	0.28
	21	0	0.56
	**Mean**	0.453333333	0.443809524
	**SD**	0.233867735	0.146757857
